# Anecdote of H. Ward Beecher

**Published:** 1888-12

**Authors:** 


### ANECDOTE OF HENRY WARD BEECHER.

It is well known that Mr. Beecher was an uncompromising advocate of woman's rights. He held that woman should be allowed to enter any sphere of usefulness to which she was intellectually, physically and educationally adapted, including the ordinary business pursuits, the lecture field and the so-called learned professions. At one of his Friday evening conferences, which were a kind of family re-union of his congregation, the propriety of participation of the female members in the discussions of the evening came up and was warmly held affirmatively in some very convincing remark by Mr. Beecher, who was immediately followed by a dull spiritless talk of unusual length, by a fair exemplar of that style of oratory. After she had succeed in wearying all present, and affording perhaps the weightiest argument that could be brought to the negative sids of the the question, Mr. Beecher arose with much deliberation, and with a face aglow with the aptness of the occasion said with inimitable drollery, "*Nevertheless*, I am in favor of allowing women to speak." It is needless to say that after this, the neverlesses had it all their own way.

